# Challenges in downstream dam passage and the effect of dam removal on Atlantic salmon ( *Salmo salar* ) smolt migrations

**DOI:** 10.1111/jfb.15770

**Published:** 2024-05-09

**Authors:** Samuel Shry, Andrew Harbicht, Hanna Forsberg, Anders Nilsson, Gustav Hellström, Martin Österling, Olle Calles

**Affiliations:** ^1^ River Ecology and Management, Department of Environmental and Life Sciences Karlstad University Karlstad Sweden; ^2^ Department of Fisheries and Oceans Moncton New Brunswick Canada; ^3^ Department of Biology—Aquatic ecology Lund University Lund Sweden; ^4^ Department of Wildlife, Fish and Environmental Studies Swedish University of Agricultural Sciences Umeå Sweden

**Keywords:** fish passage, river barriers, river restoration

## Abstract

Migration is critical for life‐cycle completion in diadromous fish species. River connectivity is vital in facilitating these large‐scale movement events, but the extent of present‐day river fragmentation can interfere with these migrations. Fish passage solutions (FPSs) are commonly implemented with the aim of improving river connectivity. In our study, we investigated the performance of two types of FPSs, spill regimes and complete dam removal, on Atlantic salmon (*Salmo salar*) smolt migrations. We used acoustic telemetry to monitor migration behavior and passage success of 120 wild smolts released in three different groups/sites: one group with two dams to pass to reach the river mouth, a second group with one dam to pass, and a control group without any barriers to pass (upstream of a recently removed hydroelectric dam). Smolt passage probabilities were similar for the two studied dams (87% and 86%) but showed variation in path choice, delay times, and loss rates. Passage success was influenced by several factors, such as body size, diel period, and water temperature, but not flow. Cumulative passage success to the river mouth was 61%, with most individuals being lost within lentic river stretches, either in the forebays of hydroelectric power stations or in naturally wide river stretches. Within the recently rehabilitated river sections (post dam removal), passage speeds were significantly faster than all other sections of the river (post‐rehabilitation x¯ = 56.1 km/day) with significantly faster speeds compared to pre‐rehabilitation (pre‐x¯ = 28.0 km/day). Our findings provide valuable information on the benefits of dam removal and highlight the need for further rehabilitation measures in upriver reaches where barriers still affect downstream passage.

## 1 INTRODUCTION

Migration is a crucial aspect of life cycles of many fish species, enabling them to access essential habitats, for example, feeding, spawning, and growth (Brönmark et al., 2014). From feeding migrations of roach (*Rutilus rutilus*) to homing and spawning of pike (*Esox lucius*) to highly mobile coastal sea trout (*Salmo trutta*), pristine river systems are migratory highways (Degerman et al., 2012; Forsman et al., 2015; L'Abée‐Lund & Vøllestad, 1987). This is especially true for fishes with complex life‐history strategies like salmonids, who may require large‐scale migrations (>100 km) to complete different life stages. Atlantic salmon (*Salmo salar*) are anadromous and iteroparous and therefore migrate between fresh water and seawater multiple times throughout their lives (Nunn & Cowx, 2012; Persson et al., 2022). After hatching, juvenile salmon spend 1–3 years in rivers growing before reacting to environmental and physiological signals to migrate to the sea, a process called smoltification (McCormick & Saunders, 1987; see, e.g., Jonsson & Jonsson, 2011 for process details). After spending 1–3 years at sea, mature adults return to their hatching river stretches to spawn. Post‐spawned salmon (kelt) can return to the sea to grow and thereafter potentially spawn multiple times throughout their life (Allan & Ritter, 1977; see, e.g., Jonsson & Jonsson, 2011 for process details). Despite the importance of restoring riverine connectivity to aid migration and natural recruitment of wild populations, most rivers remain fragmented by anthropogenic barriers (Belletti et al., 2020; Nilsson et al., 2005).

Hydroelectric power constitutes an important source of sustainable energy production, generating renewable energy with regulatory capacity. However, due to the size and complexity of hydroelectric plants (HEPs), they are also the most challenging anthropogenic barriers for fish to navigate, resulting in direct or indirect mortality, migratory delays, and reduced migratory success (Bleckmann & Zelick, 2009; Coutant & Whitney, 2000; Larinier, 2008; Nyqvist et al., 2016). Salmon smolts are particularly vulnerable to HEP barrier effects due to their small size and limited swimming capacity. HEP forebays are usually slow‐flowing, lentic environments, where smolts must successfully navigate past risks such as avian and piscine predators (Koed et al., 2006). Migration behavioral strategies in smolts have been associated with environmental factors such as diel period, flow, and water temperature (Hvidsten et al., 1995; Ibbotson et al., 2006, 2011). Length and body condition can also be important factors for migration timing, speed, and post‐smolt survival (Bohlin et al., 1996; Kallio‐Nyberg et al., 2004). Smolts have a narrow migration window, and delays caused by barriers can lead to migration being aborted, staying in the river another year (Eriksson, 1984; Jonsson & Jonsson, 2011). Today, river rehabilitation, including fish passage solutions (FPSs), is implemented to reduce such negative effects on migratory fish.

River rehabilitation refers to processes of improving the ecological health and function of a river, by, for example, improving river connectivity, spawning, and rearing habitats (Fryirs & Brierley, 2009; Smith et al., 2014 and references therein). Although the term river restoration is commonly used, it is rarely possible to fully restore a river ecosystem to its natural state, where rehabilitation comes before ecological restoration on the restorative continuum (Gann et al., 2019). Rehabilitation efforts that maintain and improve river connectivity can have significant benefits for fish populations, supporting ecosystem functions and facilitating dispersal and migration (Wohl, 2017). FPSs are a form of river rehabilitation designed to mitigate the effects of barriers by diverting fish away from the powerhouse through a bypass for safe and efficient passage, but they rarely restore complete longitudinal connectivity (Birnie‐Gauvin et al., 2019). These solutions need to be effective in both attracting fish to the bypass (attraction efficiency) and allowing them to pass the barrier (passage efficiency) (Silva et al., 2018). FPSs for upstream passage have a long history of development and implementation, whereas downstream FPSs have only recently been adopted, with few evaluated for efficiency (Calles et al., 2013, 2021; Nyqvist et al., 2017, 2018). Historically, downstream passage has been neglected, and primary passage was through the powerhouse, but today, two common downstream FPSs are spill regimes and dam removal (Katopodis & Williams, 2012). Spill regimes are set time windows of increased spill, typically through the spill gates of a HEP. Spill regimes are used to attract downstream migrating fish to and through the spill gates, and have previously been shown to be positively correlated to downstream passage success and efficiency (Ferguson et al., 2005; Scruton et al., 2008). There can, however, be significant cumulative negative effects in a multi‐barrier system, even if the FPSs are relatively efficient (Norrgård et al., 2013). The most effective rehabilitation measure for longitudinal connectivity is dam removal, which can bring the river as close as possible to its pre‐impediment state (Birnie‐Gauvin et al., 2019; Silva et al., 2018). Dam removal is increasingly common, especially for small barriers (<2 m), but even larger hydroelectric power stations have been removed in recent years where environmental concerns outweighed energy production value (Gowan et al., 2006).

Rehabilitation measures implemented to improve downstream fish passage on the River Mörrumsån, Sweden, are temporary spill regimes at the HEPs and the removal of the lowermost HEP on the river, Marieberg HEP, which occurred during the summer of 2020. Evaluating the effect of these river rehabilitation measures on smolt migration is essential for both conservation and management incentives. In this study, we use acoustic telemetry to evaluate the performance of spill regimes and dam removal for smolt passage, and address how environmental variables impact smolt migration. Our study aims to investigate smolt migration success through both modified and rehabilitated sections of the River Mörrumsån on their way to the Baltic Sea.

## 2 METHODS

The River Mörrumsån (56°09′31.1″ N 14°44′52.0″ E) located in southern Sweden is the largest river system in the region, with a catchment of 3369 km^2^, running 186 km, and a mean annual discharge of 27.3 m^3^/s. The Atlantic salmon in the River Mörrumsån constitute a genetically unique, economically important, highly productive population, which supports one of Sweden's most valuable recreational salmon fisheries. Estimated smolt production in the upriver reaches, however, decreased from approximately 21,000 in 2014 to only 3000 in 2019 (Bajinskis et al., 2020). To ensure population sustainability, decreasing mortality in the smolt migration phase is crucial. Since the removal of the lower‐most HEP, Marieberg, in August 2020, four HEPs continue to partially disrupt connectivity for migratory fish, with the fifth, uppermost HEP (Granö, around 35 km upriver from the Baltic Sea) an absolute barrier to upstream migration. The four passable dams are equipped with varying forms of fish passage solutions for both upriver and downriver passage. At lower and upper Fridafors (HEPs 3 and 4), inclined (upper) and angled (lower) guide racks divert downstream migrating fish into a bypass. These two HEPs are also equipped with nature‐like fishways for upstream migration. The FPS details of the HEPs upper and lower Hemsjö (HEPs 2 and 1) are outlined below.

**FIGURE 1:**
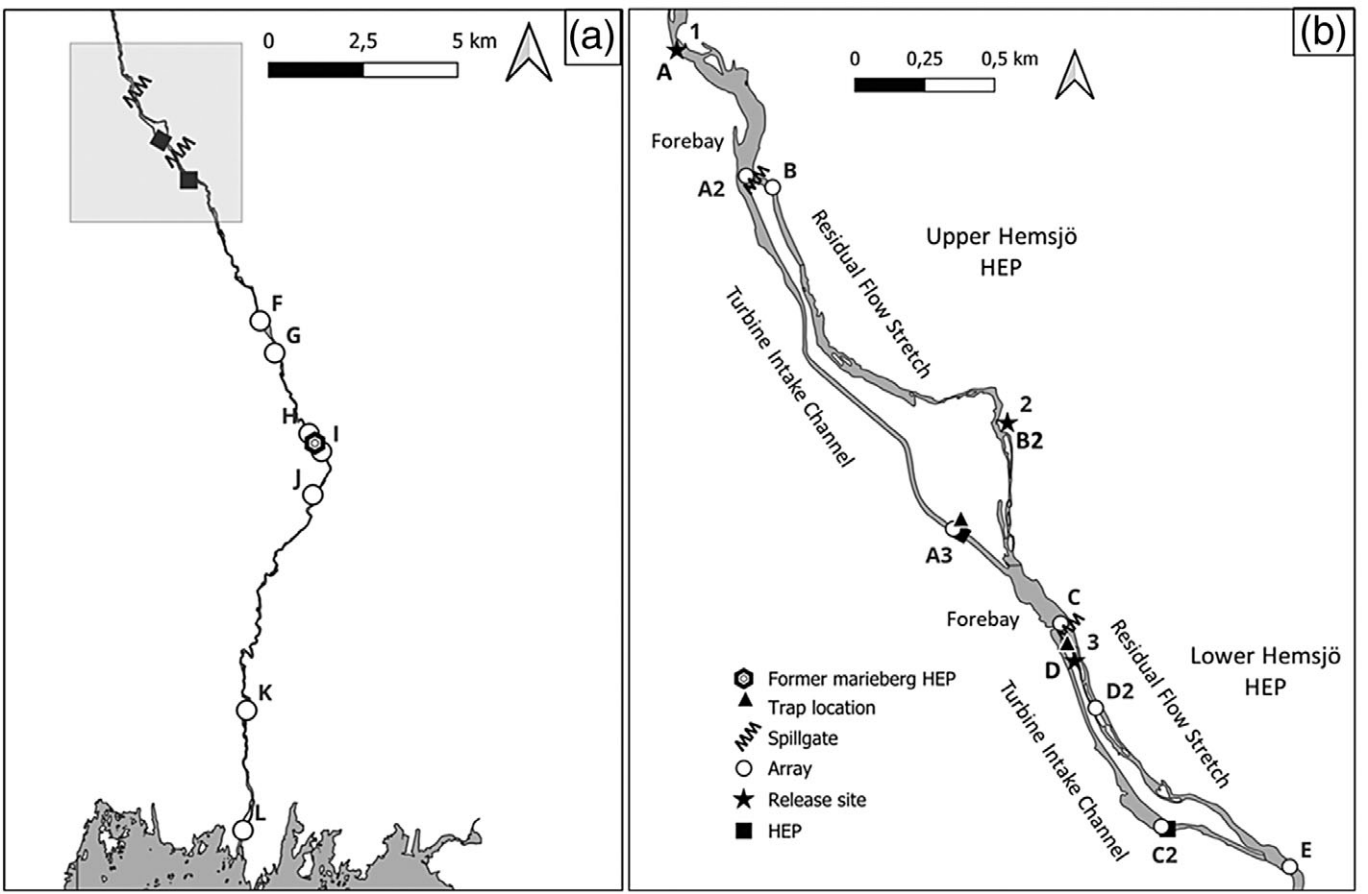
The River Mörrumsån study area and receiver locations. (a) Overview of the lower 25 km of river, indicating locations of the Hemsjö hydroelectric plants (HEPs), spill gates (zig‐zag symbols), the former Marieberg HEP (hexagon), and receiver arrays (F–L). (b) The locations of the release sites (stars, 1–3), HEPs (squares), spill gates (zig‐zag symbols), residual flow stretches, trap locations (triangles), and receiver arrays (A–E).

Upper Hemsjö HEP (HEP 2) has one powerhouse with four Francis turbines (head: 15 m, total intake capacity: 28 m^3^s^−1^) and is located at the end of a 1.4‐km‐long intake channel (Figure 1). In the forebay, there are six spill gates and a nature‐like fishway connected to the residual flow stretch. Lower Hemsjö HEP (HEP 1) has one powerhouse with one Francis turbine (head: 11.2 m, total intake capacity: 20 m^3^s^−1^) and is located at the end of an 830‐m‐long intake channel (Figure 1). In the forebay, there are five spill gates and a nature‐like fishway connected to the residual flow stretch. The forebay conditions at the Hemsjö HEPs vary in intake channel width. The intake channel (37 m wide) at upper Hemsjö starts at the spill gates and is free flowing to the powerhouse. Lower Hemsjö has a narrowed intake channel gate (intake gate width = 8 m, intake channel width = 30 m) at the spill gates, with a width one‐third of the channel width.

Downstream migrating fish can pass upper and lower Hemsjö through the intake channels and powerhouses, or through the spill gates and fishways into the residual flow stretches. To aid smolt migration, a compensatory 5‐week spill is implemented during the peak migration season in April and May. This restricts turbine intake capacity to half of the available total discharge and redirects remaining river discharge into the spill gates (Swedish Environmental Court, 2003/21–99). For upstream migration, the Hemsjö HEPs are equipped with nature‐like fishways that are located in the uppermost part of each of the residual flow stretches, parallel with the spill gates.

The former Marieberg HEP had one powerhouse with four Francis turbines (head: 4.8 m, total intake capacity: 26 m^3^s^−1^). The dam was equipped with a technical fishway (vertical slot type) for upstream migration and a similar 5‐week spill regime as the Hemsjö HEPs for downstream passage. The HEP removal restored free‐flowing river in the 3.5‐km reservoir after 102 years of damming and added another 9 km of free‐flowing river stretches, creating in total 22 km.

Salmon smolts were captured using two Wolf traps (Wolf, 1951), each located in the intake channel of the two respective Hemsjö HEPs, as described in Harbicht et al. (2021). This was the preferred method of capture to ensure the capture of smolts that had already initiated downstream migration. Traps were checked twice per day, and captured salmon smolts were checked for injuries, tagged, and released daily between April 25 and May 12, 2021. Before tagging, smolts were anaesthetized (mean sedation time 5 min 26 s, SD ± 1 min 27 s) using benzocaine (1 g benzocaine/10 mL ethanol, 0.3–0.5 mL ethanol per liter river water; Sigma‐Aldrich Sweden AB, Stockholm, Sweden), and an acoustic transmitter (V5‐1×, Innovasea, Nova Scotia, Canada) was implanted intraperitoneally via a small mid‐ventral incision. The incision was sutured using one or two stitches of monofilament suture (USP 4/0—EP 1.5, CTIgroup, Prague, Czech Republic). Transmitter weight in air corresponded to 1.6%–4.6% of individual body weight, below the recommended 8% ratio by Lacroix et al. (2004). Individual biometrics collected during the tagging procedure included length (mm), weight (g), and smolt stage based on the classification of the extent of body silvering (as described in Harbicht et al., 2021).

After initial recovery from tagging, smolt individuals were transported to their randomly assigned release sites (1, 2, and 3; Figure 1), with an even distribution from both capture sites among all release sites. Most individuals were captured in the upper Hemsjö Wolf trap (81%), with 18% (*n* = 7) possibly having to pass upper Hemsjö HEP a second time if they had not originated from the residual flow stretch between upper and lower Hemsjö HEPs. Depending on their assigned release site, fish were transported in aerated tanks filled with river water (3 × 50 L) by car between 500 m and 2.5 km (duration 1–7 min) from the tagging site. Fish were transported to their release site in groups of 1–9 individuals per tank, depending on capture date. Once at their assigned released site, fish were transferred to perforated flow‐through recovery containers. Release site 1 was located approximately 600 m upstream of the upper Hemsjö HEP spill gates and required individuals to pass two barriers (upper and lower Hemsjö HEPs). Release site 2 was located in the residual flow stretch of upper Hemsjö HEP, requiring individuals to pass one barrier (lower Hemsjö HEP). Release site 3 was located in the residual flow stretch of lower Hemsjö HEP, acting as a control group by allowing smolts to migrate through fully free‐flowing river stretches. Smolts were kept in the recovery containers until dusk, with a mean recovery time of 8 h and 23 min (SD ± 47 min). Long recovery times and dusk release were the preferred methods to ensure full recovery and improve post‐release survival (Glover & Stephen, 2023; Vollset et al., 2017). Before release, fish were observed and confirmed to be in good physical condition before being released at dusk. If individuals were recaptured in a Wolf trap, they were released downstream the trap of recapture. A total of 120 smolts were tagged and released from three release sites, approximately 40 at each site.

To monitor the route selection and downstream migration of smolts, 34 strategically placed acoustic receivers were used across the 25‐km study stretch. Receiver arrays were assigned a letter, with A being the receiver array located most upstream in the system and proceeding downstream to L at the river mouth (Figure 1). These receivers were anchored with concrete slabs, and buoys were used to maintain a vertical position about 30 cm from the riverbed. Multiple high residency receivers (HR2‐180 kHz, Innovasea) were placed in arrays at the spill gate forebays and HEP intakes of both Hemsjö HEPs, whereas all other receiver arrays comprised one receiver (VR2‐180 kHz, Innovasea) acting as a gate system (presence/absence). After initial range testing and considering the relatively narrow river width (29–61 m), it was determined that the detection efficiency of all arrays was near 100%, except for two arrays located in the residual flow stretches of each HEP, owing to the heterogeneous river morphology and lotic environment at those locations. These receiver arrays were omitted from analysis due to their low detection efficiency.

Once data were collected, raw detections were organized to create a movement table for each fish. Movement tables were used to calculate passage rates, route choices, and passage times. Covariate effects on passage outcomes were analysed using a binomial generalized linear model (GLM) to determine covariate effects on passage outcomes (success/loss). Passage rates through river sections were analysed using a Cox proportional hazards model to quantify the effect, if any, of environmental, biological, and anthropogenic covariates. The data were also compared to the results of a previous smolt migration study conducted prior to the removal of the Marieberg dam. All statistical analyses were performed using various software packages in R (R Core Team, 2023).

The movement data for each smolt were defined by the initial true detections (>2 consecutive detections) at each array, whereas passage times were calculated as the time difference between the first upstream detection and the first downstream detection for each river section delimited by the 14 array gates within the river system. The distance between each receiver gate was calculated to determine transition speeds.

To evaluate smolt passage performance in upper and lower Hemsjö HEPs, we calculated the impediment passage efficiency (*η*ip), which represents the proportion of smolts detected in the forebay that went on to successfully pass the impediment, pass via the spill gates or intake channel and represents an overall passage probability. Covariates to this model comprised release location (sites 1, 2, and 3), number of dams passed (1–2), trapping location (upper/lower Hemsjö intake), and release date (day of year). Biological covariates included total length (mm) and smolt stage (1–3). To analyse the effects of model covariates on passage probabilities we used a binomial GLM. Model selection was performed by first running a global saturated model, including all variables and two‐way interactions, then performing full subset selection and identifying the best fitting models. The most parsimonious model was selected from a subset of models within six ΔAIC of the best fitting model (Richards, 2008). This process was done using the MuMIn R package (Barton, 2016).

To evaluate passage probabilities and success, we considered environmental, anthropogenic, and biological covariates. Environmental covariates included water temperature (°C), river discharge (m^3^s^−1^), and diel period (day/night). Water temperature was obtained from each of the HR receivers every 10 min, and an average temperature (per 10 min) for each HR array was used for analysis. Discharge was measured every minute and differentiated between spill gates and the turbine intakes for each HEP (data obtained from Uniper SE). Diel periods were calculated based on sunset/sunrise times for this location during the migratory period (data obtained from SMHI). We used the closest upstream HEP as a reference for total river discharge (Granö for HEP2, HEP2 for HEP1). Anthropogenic covariates comprised release location (sites 1, 2, and 3), number of dams passed (1–2), trapping location (upper/lower Hemsjö intake), and release date (day of year). Biological covariates included total length (mm) and smolt stage (1–3).

We used time‐to‐event models to evaluate the effect of the covariates on passage rates through each river section (Castro‐Santos & Haro, 2003; Castro‐Santos & Perry, 2012). We divided the river into two sections for analysis: upper Hemsjö HEP passage and lower Hemsjö HEP passage. First true detection within in each HEP passage section (forebays) was used to assign temporally variable covariate data (water temperature, discharge, and diel period). We first fit a global model with all covariates and then used full subset selection to identify the best fitting models. The most parsimonious model was selected from among a subset of models within six ΔAIC of the best fit model (Richards, 2008). Time‐to‐event models were fit using the Survival package in R (Therneau & Lumley, 2015), while model assumptions were assessed using Schoenfeld residual plots (Therneau, 2015).

## 3 RESULTS

An initial comparative analysis of biometric conditions for each of the respective release groups revealed no statistically significant difference with regard to total length (ANOVA, *df*: 2, *F*: 0.36, *p* = 0.699, Table 1) or smolt stage (Kruskal‐Wallis, *df*: 2, *H*: 1.243, *p* = 0.537). Swimming ability and physiological condition were thus considered equal among groups.

**TABLE 1:** Biometric data for tagged Atlantic salmon smolts by release group.

Release location	Upper Hemsjö release site (1)	Lower Hemsjö release site (2)	Control release site (3)
*N* total	39	40	41
Total length (mm ± SD)	141.2 ± 11.6	140.5 ± 12.8	142.9 ± 13.9
Condition factor (*K* ± SD)	0.84 ± 0.12	0.86 ± 0.13	0.87 ± 0.10
Smolt stage (1/2/3) (%)	22/53/21	15/67/13	18/51/28

*Note*: Total number of individuals (*N*), total length (mm), condition factor (*K*), and smolt stage (1/2/3) were recorded for each fish.

The majority of the tagged smolts (*N* = 117, 97.5%) reinitiated migration after release and were successfully detected at the next receiver array downstream of their respective release locations (release site 1 = 95%, release site 2 = 98%, release site 3 = 98%). The re‐initiation of migration occurred on average 42 h after release, which resulted in the lowest speeds recorded for the entire study (release site 1: 3.85 km/day, release site 2: 6.98 km/day, and release site 3: 4.18 km/day).

Array efficiencies were calculated based on respective release location and array detections. We observed 100% detection efficiency for all arrays, except for the two residual flow stretch arrays that were omitted from analysis.

The impediment passage efficiency (*η*ip) at upper Hemsjö HEP was 86.8% (release site 1, *N* = 33 of 38), corresponding to a loss rate of 5.9% km^−1^ (Table 2). Sixty‐one percent of smolts passed through the FRC (*N* = 20), 28% passed through the power house (*N* = 13), and 5% were recaptured in the Wolf trap (*N* = 2). All 13 smolts passing upper Hemsjö via the powerhouse survived (*η*_turbine_ = 100%), with losses occurring either in the forebay reservoir (*N* = 4) or as smolts navigated the residual flow stretch (*N* = 1) (Figure 1). Navigation time through the upper Hemsjö forebay was faster for individuals entering the residual flow stretch than for those entering the turbine intake channel (Mann–Whitney, *U*: 63.00, *Z*: −2.469, *p*‐value: 0.013). However, route‐specific differences in migration time were non‐significant (Mann–Whitney, *U*: 66.00, *Z*: −1.817, *p* = 0.072).

**TABLE 2:** Summary of Atlantic salmon smolt passage counts, broken down by location and route selection (powerhouse, spill gates).

Location		Passage route	Arrive	Pass	Passage rate (%)
Upper Hemsjö (HEP 2)	Release site 1		39		
	Total passage		38	33	86.8
		Powerhouse	13	13	100
		Spill gates + FRC	25	20	80
	From release site 1 (survivors)		33		
Lower Hemsjö (HEP 1)	Release site 2		40		
	Total passage		72 (33/39)	62 (28/34)	86.1 (87.5/87.2)
		Powerhouse	34 (11/23)	28 (8/20)	82.4 (72.7/87.0)
		Spill gates + FRC	38 (22/16)	34 (20/14)	89.5 (90.9/87.5)
	From release site 1 + 2 (survivors)		62		
	Release site 3		41		
Marieberg/control	Passage H–I		85 (24/28/33)	84 (24/28/32)	98.8 (100/100/96.9)
River mouth			71 (20/24/27)		61.1 (52.6/61.5/69.2)

*Note*: For each location, counts are divided by initial detection (arrive), detection after successful passage (pass), and the proportion of surviving individuals per location (passage success).

Abbreviation: HEP, hydroelectric project.

Passage efficiency for lower Hemsjö was very similar to that for upper Hemsjö, for both release groups (i.e., release site 1 and release site 2), with an impediment passage efficiency (*η*ip) of 86.1% (*N* = 62 of 72), corresponding to a loss rate of 10.5% km^−1^. Sixty percent of these individuals were lost within a 600‐m section of the river between the powerhouse intake channel and 500 m downstream, corresponding to a loss rate of 36% km^−1^ in this river section. Fifty‐five percent of smolts passed through the residual flow stretch (*N* = 34), with 45% passing through the power house (*N* = 28). Route selection was significantly different between release sites (*X* ^2^: 7.283, *df*: 1, *p* = 0.007) with fish from release site 1 (i.e., fish that successfully passed HEP2) migrated primarily through the spill gates (67%) and release site 2 (i.e., fish without any previous HEP passage experience) migrating primarily through the powerhouse (59%). There were 11 individuals that migrated through both residual flow stretches (release group 1) and only three individuals that migrated through both powerhouses. Forebay navigation through lower Hemsjö was significantly different depending on their route selection, similar to what was observed for upper Hemsjö, with faster forebay navigation for individuals entering the residual flow stretch than those entering the turbine intake channel (Mann Whitney *U*: *p* < 0.001). There were, however, no observed significant differences in passage times between passage routes (Mann Whitney *U*: *p* = 0.364) or between release sites (Mann Whitney *U*: *p* = 0.972).

Passage probabilities were not significantly affected by covariate effects at either of the two Hemsjö HEPs. After subset selection, the upper Hemsjö HEP global model gave three best fitting models under the six ΔAIC thresholds: release date, trap location, and the null model (Table 3). None of the covariates had a significant effect on passage probability, and as the null model was within six ΔAIC of the best, it was the most parsimonious. All smolts had the same passage probability at upper Hemsjö HEP, that is, 86.8%, regardless of any covariates.

**TABLE 3:** Covariates with passage probabilities at upper Hemsjö HEP.

Model	*K*	Log likelihood	AIC	ΔAIC
Release date	1	−12.17	28.71	0
Trap location	1	−13.33	31.02	2.31
Null	0	−14.59	31.12	2.41

*Note*: Results from the full subset selection of the global model with a six ΔAIC threshold.

Abbreviation: HEP, hydroelectric project.

After subset selection of the lower Hemsjö HEP model, diel period and the null model were identified as the only models within six ΔAIC units (Table 4). Diel period was within six ΔAIC of the null model, making the null model most parsimonious. All smolts had the same passage probability at lower Hemsjö HEP, that is, 86.1%, regardless of any covariates.

**TABLE 4:** Covariates with passage probabilities at lower Hemsjö HEP.

Model	*K*	Log likelihood	AIC	ΔAIC
Diel period	1	−27.09	58.38	0
Null	0	−28.39	58.85	0.46

*Note*: Results from the full subset selection of the global model with a six ΔAIC threshold.

Abbreviation: HEP, hydroelectric project.

When running the time‐to‐event model, we found that passage rates for the two Hemsjö HEPs were influenced by water temperature, diel period, and total smolt length. For upper Hemsjö, the best‐fit model, with no other models within six ΔAIC, showed that diel period and water temperature influenced passage rates (Table 5). During the day, smolt passage rates more than doubled (114% increase) when water temperatures increased by 2.6°C (1SD) over the mean. Furthermore, smolt passage rates increased fourfold (402% increase) at night relative to during the day.

**TABLE 5:** Subset of models within six ΔAIC of the best‐fitting time‐to‐event model for smolt transitions through the upper Hemsjö HEP section of the River Mörrumsån.

		Models < six ΔAIC			Best‐fit model			
Model parameters	*K*	Log likelihood	AIC	ΔAIC	Variable	HR	95% CI	*p*‐Value
Water temperature + diel period	2	−69.47	143.37	0	Temperature	2.14	1.35, 3.40	<0.01
Null	0	−78.09	156.18	12.80	Night	5.02	1.51, 16.6	<0.01

Abbreviation: HEP, hydroelectric project.

For lower Hemsjö HEP, the best‐fit model included both diel period and total length, though the more parsimonious null model was within six ΔAIC (Table 6). According to the best‐fit model, an increase in smolt length of 11 mm (1SD) over the mean (140 mm) increased the passage rate by 35%. Smolt passage rates were up to three times faster at night relative to during the day, but the extent of this effect should also be noted (95% CI = 0.98, 4.07; *p* = 0.056).

**TABLE 6:** Subset of models within six ΔAIC of the best‐fitting time‐to‐event model for smolt transitions through the lower Hemsjö HEP section of the River Mörrumsån.

Model parameters	Models < six ΔAIC				Best‐fit model			
	*K*	Log likelihood	AIC	ΔAIC	Variable	HR	95% CI	*p*‐Value
Length + diel period	2	−176.42	357.06	0	Length	1.35	1.07, 1.69	0.010
Length	1	−178.37	358.82	1.75	Night	2.00	0.98, 4.07	0.056
Diel period	1	−179.25	360.59	3.52				
Intercept	0	−180.45	360.91	3.84				

Abbreviation: HEP, hydroelectric project.

**FIGURE 2:**
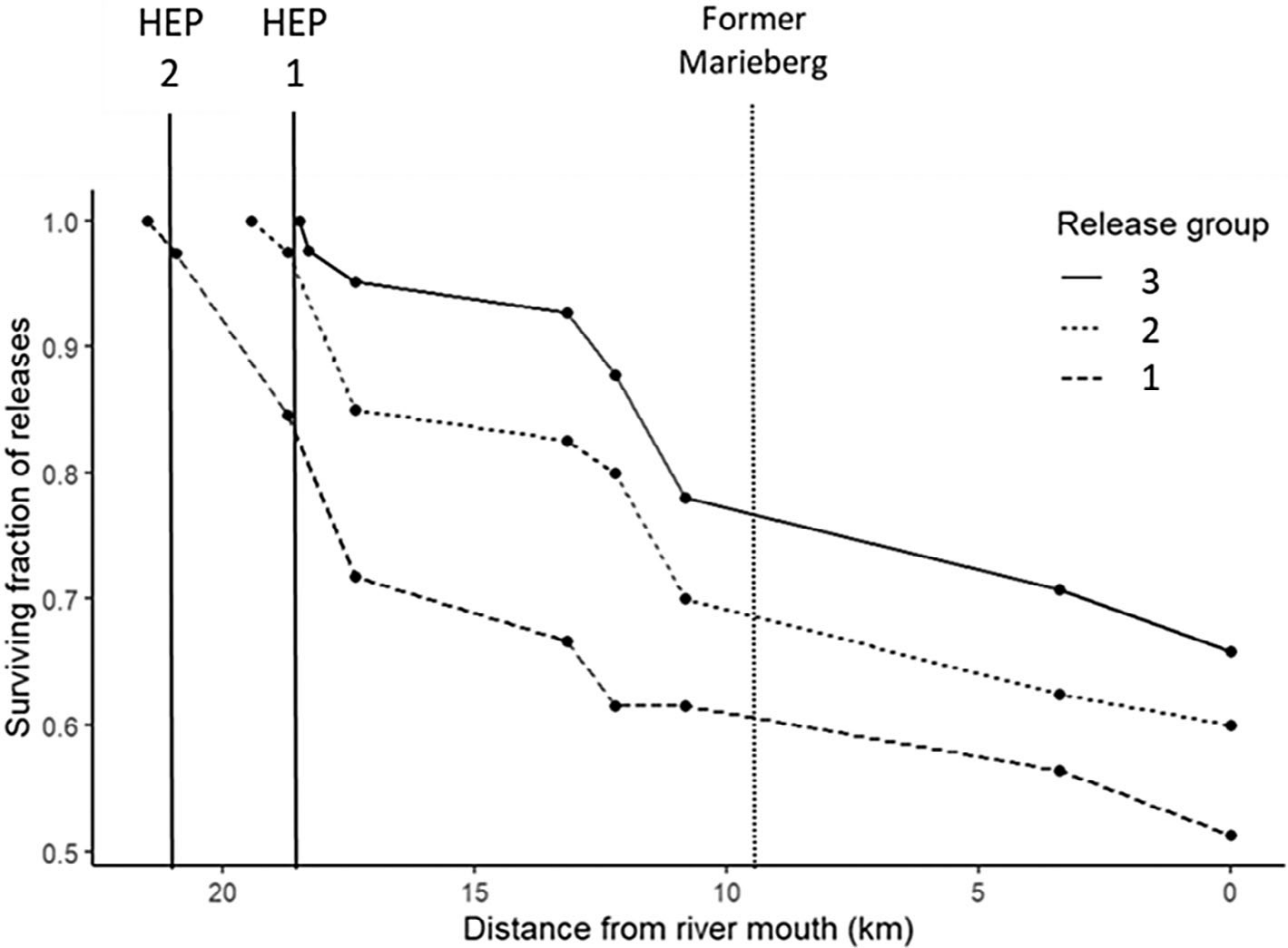
Cumulative passage success from release to the river mouth for each release group (release group 3: solid line, release group 2: dotted line, release group 1: dashed line). Locations of each hydroelectric plant (HEP) are also highlighted in relation to their distance from the river mouth (HEP 2: upper Hemsjö, HEP 1: lower Hemsjö, and the removed Marieberg HEP).

Passage success to sea, that is, smolts that reinitiated migration and were detected at the last array, around 500 m from the river mouth, was 61% (*N* = 71; Figure 2) without any significant difference in proportions of successful passage between release groups (release site 1 = 52.6%, release site 2 = 61.5%, release site 3 = 69.2%; Kruskal‐Wallis, *X* ^2^: 2.21, *df*: 2, *p* = 0.33; Figure 2). This migration from their relative release sites to the river mouth took on average 3.6 days (range = 1.1–10.1, SD = 2.7), corresponding to migration speeds of 30.1 km/day for release site 1 (25.3 km), 29.5 km/day for release site 2 (23.2 km), and 30 km/day for release site 3 (22.4 km). The remaining smolts (*N* = 49) did not exit the river during the battery life of the tag (~27 days). These individuals were assumed to be lost due to predation as losses were accrued across each migratory stretch to the sea (Figure 2).

**FIGURE 3:**
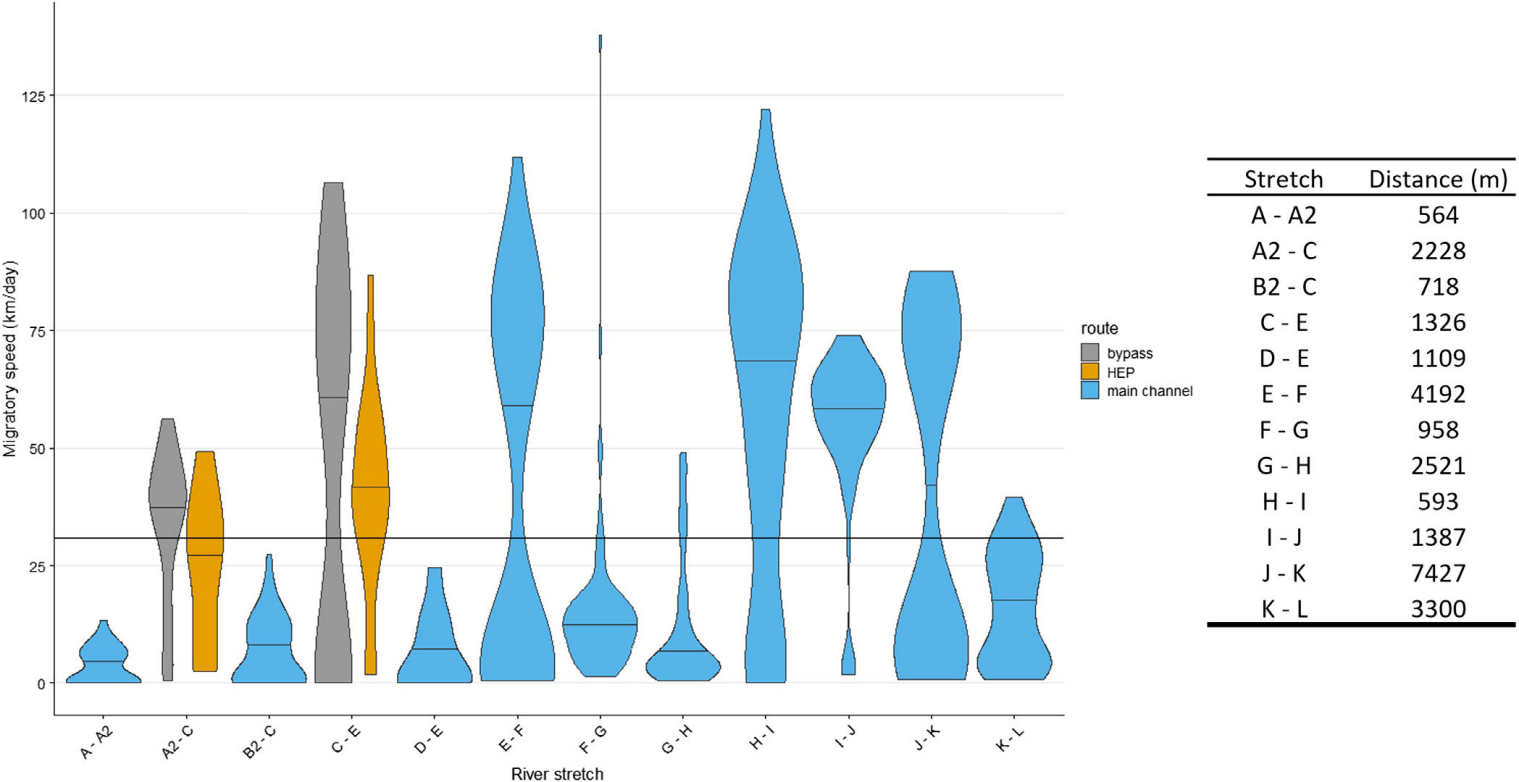
Smolt speeds for each river stretch from release to the river mouth. Color indicates river passage type (free‐flowing main channel (blue), turbine intake and hydroelectric plant [HEP] powerhouse (orange), or HEP bypass and residual flow stretch (grey)). Median lines indicated for each river section as well as a mean line of overall migration speed. Table indicates river stretch distances in meters.

Some of the slowest migration speeds were recorded through lentic, slow‐flowing stretches for all release groups (F‐G and G‐H averaged; release site 1: 16.3 km/day, release site 2: 10.9 km/day, release site 3: 13.4 km/day; Figure 3), and as smolts migrated through the river mouth (K‐L; release site 1 = 16.8 km/day, release site 2 = 16.1 km/day, release site 3 = 16.4 km/day). Migration losses over free‐flowing kilometers varied between 0% and 5.4% km^−1^, (x¯ = 2.1%), with the highest losses recorded through the lentic environments (F‐G; 5.4% km^−1^).

One individual was lost navigating the former Marieberg HEP section, and therefore the post‐removal passage efficiency was 99%, with all release groups represented (release site 1, release site 2, and release site 3). Post‐passage rates through the river section directly downstream the former powerhouse (I–J) were 100%. Pre‐rehabilitation, two individuals were lost in the same sections of the river at Marieberg HEP (Harbicht et al., 2021). One individual was lost in the forebay, with another lost directly after passage through the former HEP, reflecting an impediment passage efficiency of 96% and a pre‐passage rate of 96% directly downstream the former HEP. Speeds through the former HEP section were significantly faster than any other river sections (receiver array H‐I x¯: 57.4 km/day, receiver array I‐J x¯ = 54.9 km/day; ANOVA, *df*: 2, *F*: 44.5, *p* < 0.001; Figure 3). Compared to pre‐rehabilitation, speeds through the former Marieberg HEP river sections were significantly faster post‐rehabilitation (pre‐x¯ = 28.0 km/day, post‐x¯ = 56.1 km/day, Mann Whitney *U*: *p* < 0.001).

## 4 DISCUSSION

Our study found that downstream migration for smolts in the lower 25 km of the river Mörrumsån is challenging. Impediment passage efficiencies of 86%–87% were recorded for both Hemsjö HEPs with around half of the individuals using the downstream FPS surface spill, and therefore further actions are recommended to improve downstream passage success at these sites. However, the removal of Marieberg HEP has improved passage conditions and eliminated delays, improving overall migratory success to sea.

The impediment passage efficiencies of 86%–87% at upper and lower Hemsjö HEPs were below the recommended passage efficiency of >90% for any HEP (Calles et al., 2013; Silva et al., 2018). Interestingly, the highest losses were observed at different locations for the two HEPs. At Upper Hemsjö, most losses occurred in the forebay, whereas at lower Hemsjö, losses were observed in both the forebay and within the intake channel and powerhouse. It can, however, be difficult to differentiate fish losses in the forebay from mortality induced by turbine passage. Fish losses in these sections are likely due to both direct and indirect effects of HEP passage (Coutant & Whitney, 2000; Ferguson et al., 2011; Larinier, 2008). Dead Atlantic salmon smolts have previously been found to drift up to 2.4 km downstream, but determining the exact location and cause of mortality is difficult to assess (Havn et al., 2017). Results from the Hemsjö HEPs are consistent with those of other studies reporting high losses in HEP forebays, where low water velocities make navigation difficult, and predation poses an additional challenge (Hinch et al., 2022; Venditti et al., 2000). Disorientation in the forebays can lead to increased passage time, which often leads to increased predation in these areas (Coutant & Whitney, 2000; Larinier, 2008; Nyqvist et al., 2016). We found that fish were not significantly delayed in the HEP forebays, but even so, HEP passage loss rates accounted for 49% of total river losses for the two HEP passage release groups. If fish migrate through the powerhouse, rack impingement, blade‐strikes, and sudden pressure changes can induce mortality, with mortality rates between 5% and over 90% for Francis turbines (Larinier, 2008). Of the fish lost between the lower Hemsjö HEP intake channel and 500 m downriver, we could not determine exactly where or how these individuals were lost, but we have found this area to have high loss rates and pose a potential bottleneck for smolt migration. Indirect delayed mortality from HEP passage has been observed in other studies (Ammar et al., 2020; Ferguson et al., 2011), but we were unable to detect any differences in downstream migratory success among different HEP passage routes or between HEP passage groups and the control group. Because of the relatively low loss rate in the river section downstream lower Hemsjö HEP (array E–F, rate = 0.094%, distance = 4.2 km), it is unlikely that direct mortality occurred from HEP passage. Thirty‐one of the 71 individuals that were detected at the river mouth had successfully passed through at least one HEP powerhouse, with two of these individuals passing through both HEPs. Additionally, we did not observe any significant covariate effects on passage probabilities. It appears that individuals were able to pass the HEP sections similarly regardless of prevailing environmental conditions, capture location, and release location. As the smolt migration period takes place over a short time window, environmental conditions were relativity stable throughout the study.

Based on the time‐to‐event analysis, water temperature, diel period, and smolt total body length influenced HEP passage. At upper Hemsjö, increased water temperature and nocturnal migration significantly increased passage rates. At lower Hemsjö, increasing fish length and nocturnal migration increased passage rates significantly and nearly significantly, respectively. Nocturnal migration has previously been found to positively affect passage rates, as visual predation is reduced at night (Aarestrup et al., 2014; Scruton et al., 2007). The positive relationship between temperature and passage is also commonly found in other studies; higher temperature increases fish activity and acts as one of the major cues for smolt migration, and has been previously linked to increased daytime migratory behavior (Haraldstad et al., 2017; Hembrel et al., 2001; Jonsson & Ruud‐Hansen, 1985). At lower Hemsjö, the null model was most parsimonious at the six ΔAIC limit, but body length and diel period were also found to have important effects on passage success. If we were to interpret these results with a three ΔAIC limit, then the most parsimonious model for lower Hemsjö would be body length and diel period. Body length is commonly an important factor in smolt passage studies, where larger individuals tend to have higher passage success and return rates, but also higher turbine‐induced mortality (Armstrong et al., 2018; Beckman et al., 1998; Rivinoja, 2005). We also found that passage during the night hours increased passage success at lower Hemsjö HEP, similar to upper Hemsjö HEP, but had a lower effect (upper Hemsjö = 402% increase, lower Hemsjö = 100% increase).

Passage efficiency at the former Marieberg HEP was higher post‐rehabilitation (99%) than what was observed pre‐rehabilitation (82%, Calles et al., 2013; 86%, Harbicht et al., 2021). Only one individual was lost within the former Marieberg HEP forebay and powerhouse river sections. Passage rates and speeds through these river sections (receiver arrays H‐I = 57.4 km/day and I‐J = 54.9 km/day) were higher than any other river section, suggesting that rehabilitation has improved passage success. The obvious physical effects of the dam structure being removed could have made passage faster and easier, with no obstacles to movement downstream and higher flow velocities after removal. For example, Harbicht et al. (2021) found a correlation between migration speed and river width, with slower speeds occurring through naturally wide river sections and in the former Marieberg HEP reservoir. Slower migration speeds through lentic environments were also observed, in this study and others, as standing water, both natural and man‐made, can disrupt smolt orientation and flow velocity ques (Honkanen et al., 2021; Thorpe et al., 1981). Pre‐removal studies found no direct delays from Marieberg HEP, and losses were attributed to predation in the forebay (Calles et al., 2013; Harbicht et al., 2021). Other studies have also found increased smolt passage rates post dam removal rehabilitation (Birnie‐Gauvin et al., 2018; Stich, Bailey, et al., 2015; Stich, Kinnison, et al., 2015). The lack of a forebay and dam structure to navigate seems to have re‐created necessary conditions for faster passage and lower loss rates, contributing to an increased full‐river passage success post dam removal.

Our results indicate that approximately 61% of tagged individuals survived to reach the sea, with an average loss rate of 2.1% km^−1^. The control release group (release site 3) showed the highest full‐river passage success rate (66%), which is almost double the 36% passage rate reported by Harbicht et al. (2021) for the same stretch of river pre‐dam removal. Total smolt survival recorded in this study is relatively high compared to other systems even with passage through two HEPs (Chavarie et al., 2022; Flávio et al., 2021; Lothian et al., 2018). As these results derive from only 2 years of data, multi‐year smolt migration studies would enhance the understanding of temporal effects in this system (Chaput et al., 2019; Jensen et al., 2012). Similar to migration speeds, most fish losses occurred in lentic environments, during passage at the two Hemsjö HEP forebays and between receiver arrays F‐G, where the river naturally widens into a 0.8‐km lentic section. It is not unexpected to observe higher losses at the HEPs due to barrier passage difficulties (Aarestrup & Koed, 2003; Calles & Greenberg, 2009; Nyqvist et al., 2017), and increased mortality has been previously associated with river width in this system (Harbicht et al., 2021). Passage losses in free‐flowing river sections (0%–5.4% km^−1^) were similar to those in other studies (Holbrook et al., 2011; Norrgård et al., 2013). Overall, there was a negative relationship between river migration distance and passage success. In fragmented rivers, spawning habitat rehabilitation in lower river reaches can hereby be an important factor for improving both spawning prerequisites and smolt downstream migration success (Hill et al., 2019). As our study design focused on the riverine passage, we did not study estuary transition zone passage and early marine mortality, areas that have previously been found to be bottlenecks to smolt migratory success (Flávio et al., 2020; Thorstad et al., 2012). Further studies should investigate estuarial and early marine phase passage rates to have a holistic picture of survival at sea.

We have found that reestablishing longitudinal connectivity via dam removal has had positive effects for downstream smolt migrations by improving passage speed and overall smolt passage success in the lower reaches of the River Mörrumsån. However, we found that passage success is still not optimal through the Hemsjö HEPs, even though we did not find significant differences in migratory success between release groups. We found that the 5‐week remedial spill successfully guided between 55% and 60% of smolts away from the turbine intake channel, and we recommend that additional downstream fish passage solutions be designed and implemented, perhaps in combination with the existing remedial measures. We believe the Marieberg dam removal not only benefited smolt migration but also adult spawning migration runs. Marieberg HEP was assumed to have hindered a proportion of spawners from migrating upstream for spawning, even though no quantitative evaluations of passage performance were ever carried out. From boat electrofishing surveys conducted before and after dam removal (unpublished data), there has been an increase in the number of adult spawners in the previously inundated stretch upstream the former dam, providing evidence for increased spawning runs higher in the system, that will likely improve spawning success and future parr densities.

Overall, this study emphasizes the importance of a holistic river management plan for fish passage. In fragmented systems, telemetry can provide important information about passage rates, speeds, route selection, and loss rates. We hope that this information on cumulative effects on downstream fish passage success in the River Mörrumsån will be used to direct actions to further increase fish passage success. This study enhances our understanding of smolt migrations in this system and emphasizes the importance of river rehabilitation efforts in mitigating anthropogenic impacts on wild fish populations.

## AUTHOR CONTRIBUTIONS

Conceptualization and study design: Olle Calles, Samuel Shry, P. Anders Nilsson, Gustav Hellström, and Martin Österling. Data generation: Hanna Forsberg, Samuel Shry, Andrew Harbicht, and Gustav Hellström. Data analysis: Andrew Harbicht, Samuel Shry, and Hanna Forsberg. Manuscript preparation: Andrew Harbicht, Samuel Shry, Hanna Forsberg, Olle Calles, P. Anders Nilsson, and Martin Österling. Funding: Olle Calles and Martin Österling.
